# Nanodomain Clustering of the Plant Protein Remorin by Solid-State NMR

**DOI:** 10.3389/fmolb.2019.00107

**Published:** 2019-10-15

**Authors:** Anthony Legrand, Denis Martinez, Axelle Grélard, Melanie Berbon, Estelle Morvan, Arpita Tawani, Antoine Loquet, Sébastien Mongrand, Birgit Habenstein

**Affiliations:** ^1^Institute of Chemistry & Biology of Membranes & Nanoobjects (UMR5248 CBMN), IECB, CNRS, Université Bordeaux, Institut Polytechnique Bordeaux, Pessac, France; ^2^Laboratoire de Biogenèse Membranaire - UMR 5200 - CNRS, Université de Bordeaux, Villenave-d'Ornon, France; ^3^European Institute of Chemistry and Biology - UMS3033/US001, Pessac, France

**Keywords:** nanodomains, lipid raft, solid-state NMR, membrane protein, plant protein, phosphoinositide, sterol, remorin

## Abstract

Nanodomains are dynamic membrane subcompartments, enriched in specific lipid, and protein components that act as functional platforms to manage an abundance of cellular processes. The remorin protein of plants is a well-established nanodomain marker and widely serves as a paradigm to study nanodomain clustering. Located at the inner leaflet of the plasma membrane, remorins perform essential functions during signaling. Using deuterium and phosphorus solid-state NMR, we inquire on the molecular determinants of the lipid-protein and protein-protein interactions driving nanodomain clustering. By monitoring thermotropism properties, lipid acyl chain order and membrane thickness, we report the effects of phosphoinositides and sterols on the interaction of various remorin peptides and protein constructs with the membrane. We probed several critical residues involved in this interaction and the involvement of the coiled-coil homo-oligomerisation domain into the formation of remorin nanodomains. We trace the essential role of the pH in nanodomain clustering based on anionic lipids such as phosphoinositides. Our results reveal a complex interplay between specific remorin residues and domains, the environmental pH and their resulting effects on the lipid dynamics for phosphoinositide-enriched membranes.

## Introduction

The fluid mosaic model of Singer and Nicolson (1972) describes a biological membrane as a bilayer of phospholipids, hydrophobic parts buried and hydrophilic parts exposed, with membrane proteins spanning or anchoring to the bilayer. In this model, lipid heterogeneity, and precise lipid-protein interactions that might lead to cooperative local enrichment of specific components, i.e., nanodomain organization becomes conceivable. Due to their eclectic composition, membranes are key players in cell biology, and have a plethora of functions (Grecco et al., 2011; Ott, 2017), thus constituting prime drug design targets.

The lipid raft hypothesis, that is the lateral segregation of membrane components, was first formulated by Simons and Ikonen (1997). Lipid rafts have different characteristics depending on the organism and bore different names throughout past decades, accounting for the variety of techniques allowing their detection. Here, we will commit to the word nanodomain because remorin, isoform *St*REM1.3, segregates into around 80 nm wide domains (Raffaele et al., 2009). Considering the recent technological advancements in analyzing nanodomains (Sezgin et al., 2017), the description given by Pike (2006), herein named “membrane raft,” is still relevant: a small (10–200 nm wide) region of a membrane, not necessarily the plasma membrane (PM), enriched in sterols, and specific proteins and lipids, like phosphoinositides (PIPs) (Furt et al., 2010; Brown, 2017). Nanodomain formation thus relies on the diffusion of the membrane components creating detectable membrane heterogeneity. Importantly, the diffusing, internally ordered lipids can separate into liquid-ordered (Lo) and liquid-disordered (Ld) regions in membranes, including PM-mimicking environments (Kaiser et al., 2009). Considering typical nanodomain compositions, these membrane regions should have a tendency of manifesting Lo behavior, usually containing lipids fostering liquid order such as sterols.

Members of the multigenic, aerial plant-specific family of remorins (Raffaele et al., 2007) are well-known protein markers of such nanodomains in plants (Mongrand et al., 2004; Raffaele et al., 2009). This family is made of 6 phylogenetic groups sharing a canonical C-terminal domain, containing a segment with high coiled-coil propensity, and a variable N-terminal domain (Raffaele et al., 2007). Our study will focus on potato (*Solanum tuberosum*) remorin group 1 isoform 3 (*St*REM1.3). It contains an intrinsically disordered N-terminal domain involved in protein-protein interactions (Raffaele et al., 2009) and phosphorylation events (Marín et al., 2012; Perraki et al., 2018), a coiled-coil domain involved in homo-trimerisation (Martinez et al., 2018) and a C-terminal domain called the Remorin C-terminal membrane Anchor (RemCA).

*St*REM1.3 specifically binds negatively charged liposomes *in vitro*, as we showed in Perraki et al. (2012). Expression of a plasma membrane (PM)-targeted phosphatidylinositol 4-phosphatase MAP-SAC1p in *Nicotiana benthamiana* leaves leads to a strong decrease of a *St*REM1.3's PM targeting and lateral segregation, implying that it has a specific affinity for PI4P of the PM's inner leaflet (Gronnier et al., 2017). Moreover, PM vesicles of *Nicotiana benthamiana* leaves treated with methyl β-cyclodextrin, a chelator of sterols, showed a complete loss of remorin nanoclustering, indicating phytosterols also play a role in the formation of nanodomains (Raffaele et al., 2009). Similarly, treatment with sterol inhibitor fenpropimorph strongly impaired nanodomain clustering, without affecting PM targeting of StREM1.3 (Gronnier et al., 2017).

**Figure 1:**
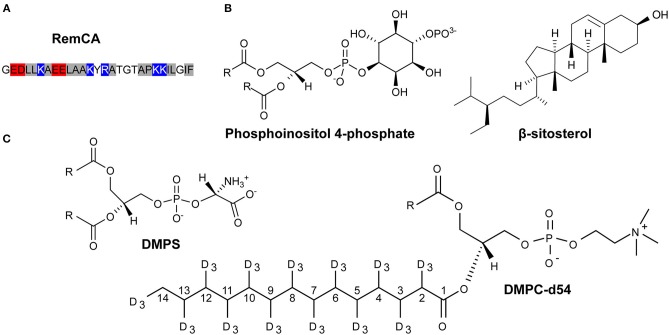
Primary sequence of RemCA (red: negatively charged residues, blue: positively charged residues, hydrophobic residues: gray **(A)** and lipids used in this study **(B,C)**, for the sake of clarity, carbon positions along the acyl chain of DMPC-d54 are explicitly labeled.

Membrane anchoring of *St*REM1.3 proceeds over an unconventional mechanism with a hydrophilic domain tethering to the membrane and organizing into nanodomains (Gronnier et al., 2017). From the latter study, we formulated a hypothetical two-step mechanism for the formation of *St*REM1.3 nanodomains: (1) the remorin, likely as a homotrimer (Perraki et al., 2012), binds to PI4P of the PM's inner leaflet with a ratio of 1 PI4P moiety per 1 RemCA, therefore clustering the 3 lipids with the remorin; (2) PI4P moieties, bearing mostly saturated acyl chains (Furt et al., 2010), will preferentially attract plant sterols, of which sitosterol is the main representative, while other PI4P moieties gather around the nascent PI4P nanocluster (Picas et al., 2016). Removal of sterols by m-β-cyclodextrin on PM vesicles disrupts nanodomain clustering but not PM binding (Raffaele et al., 2009), revealing a dependency of nanodomain formation on sterols. Remorins are also known to form filaments *in vitro* though no such structure has yet been unambiguously observed *in vivo* (Bariola et al., 2004; Martinez et al., 2018). In essence, remorins might bind over a complex mechanism involving electrostatic interactions between the positively charged lysines in the RemCA (Figure 1A) with negatively charged PI4P head groups (Gronnier et al., 2017), favoring the formation of sitosterol and PIP enriched clusters (Figure 1B).

Structural studies of the mechanisms behind protein-lipid nanodomain formation and assembly at the atomic level remain scarce, mostly relying on molecular dynamics (Ackerman and Feigenson, 2015; Gronnier et al., 2017; Javanainen et al., 2017). Indeed, the size, insolubility, non-crystallinity and required native state of the objects of interest disqualify most biophysical techniques, such as X-ray crystallography and liquid-state NMR, to obtain experimental evidence at the atomic level. We use solid-state NMR spectroscopy (ssNMR), a technique tailored to study such objects, both in terms of protein fold and interactions (Habenstein and Loquet, 2016; Loquet et al., 2017) in the native membrane environment (Cady et al., 2010; Baker et al., 2015; Stanek et al., 2016; Ladizhansky, 2017; Lalli et al., 2017; Mandala et al., 2018) and to determine membrane dynamics and interactions at the atomic level using static deuterium NMR on deuterated liposomes (Dufourc et al., 1992; Beck et al., 2007; Huster, 2014; Yao and Hong, 2014; Molugu et al., 2017).

Here, we aim at deciphering the molecular mechanisms by which *St*REM1.3 anchors to PM to form nanodomains using deuterated liposomes along with deuterium (^2^H) ssNMR as our main tool. To reflect the lipid-protein interactions and dynamic behavior during nanodomain formation, we chose to work with liposomes of nanodomain-mimicking lipid compositions, including PIPs and sitosterol (Figure 1B). Using different remorin constructs including the membrane anchor RemCA alone, in the wild-type and several mutated versions, Rem_86−198_, composed of RemCA and the coiled-coil segment, and the intact *St*REM1.3, we monitored the overall mechanisms and the fine molecular implications of nanodomain assembly guided by remorin. We tested the behavior of RemCA and its critical mutants in various nanodomain-like and unlike lipid environments, assessing the lipid preferences and electrostatic interactions involved in the anchor-membrane association. We then monitored the impact of the coiled-coil trimerisation domain, and its capacity of forming higher-order oligomers, as well as of the intrinsically disordered domain (IDD) of *St*REM1.3 on its membrane-binding behavior.

## Materials and Methods

### Protein Production and Purification

Synthetic RemCA peptides were ordered from GenScript HK Limited at >90% purity with N-terminal acetylation.

*Escherichia coli* BL21-DE3 were transformed with a pET24 vector containing the DNA encoding for *St*REM1.3 or REM_86−198_ and plated onto LB-agar plates containing 30 μg/mL kanamycin. A pre-culture of 100 mL LB medium was inoculated with a single transformed colony and incubated at 37°C overnight (about 20 h). 1L of LB medium is inoculated with the pre-culture at OD_600_ = 0.2 and incubated at 37°C until OD_600_ = 0.7–0.8. Protein production was induced with 1 mM of IPTG at 18°C overnight. Cells were pelleted at 6,000 g for 20 min at 4°C and resuspended in a lysis buffer (20 mM HEPES, 150 mM NaCl, 20 mM imidazole, 1 mM PMSF, 0.02% NaN_3_, pH = 7.4) containing protease inhibitors (Complete, Roche). Cells were sonicated on ice at 30% magnitude three times (30s on, 30s off) and centrifuged at 15,000 g for 30 min at 4°C to recover the supernatant.

Purification of *St*REM1.3 was achieved with an Akta Pure 25 HPLC system (GE Healthcare) on a HisTrap affinity column equilibrated in wash buffer (20 mM HEPES, 150 mM NaCl, 20 mM imidazole, 0.02% NaN_3_, pH = 7.4). The protein was eluted with a stepwise gradient of elution buffer (20 mM HEPES, 150 mM NaCl, 500 mM imidazole, 0.02% NaN_3_, pH = 7.4): 15, 40, 80, and 100% elution buffer. About 20 mg of pure unlabelled *St*REM1.3 were obtained from a 1L culture in LB medium. Purification of REM_86−198_ was performed as described previously in Martinez et al. (2018). After purification, proteins were buffer-exchanged against a reconstitution buffer (10 mM HEPES, 10 mM NaCl, 0.02% NaN_3_, pH = 7.4) using a HiPrep gel filtration column (GE Healthcare).

### Liposome Reconstitution

1,2-dimyristoyl-d54-sn-glycero-3-phosphocholine (DMPC-d54), 1,2-dimyristoyl-sn-glycero-3-phosphocholine (DMPC), 1,2-dimyristoyl-sn-glycero-3-phosphatidylserine (DMPS), β-sitosterol were purchased from Avanti Polar Lipids, Inc. (USA) and phosphoinositides from bovine brain (PIP mix, PS/PI/PI4P/PI(4,5)P_2_ 50/20/15/15 (mol/mol) from Sigma-Aldrich.

Synthetic RemCA peptides were co-solubilized in CHCl_3_/MeOH 2/1 (V/V) with the appropriate amount of lipids (peptide/lipid molar ratio of 1/20). Organic solvents were evaporated under an air stream, hydrated, and then lyophilised. Lipid and lipid-peptide powders were rehydrated with deuterium-depleted water or 260 mM Tris buffer. The hydrated samples were submitted to three freeze-thaw-vortex cycles (1 min in liquid N_2_, 10 min at ~40°C, 20 s vortexing) for sample homogenization and packed into a ssNMR rotor. This sample preparation has been used for triplicate error assessment on liposomes of the following composition: PC/β-sitosterol/PIP mix 75/15/10 (molar ratio) and RemCA K192A/K193A (lipid/peptide = 20) (Figures S1A,C,E).

For protein reconstitution, pre-formed liposomes were incubated overnight at 30°C with the protein at a protein/lipid molar ratio of 1/20. The milky solution was centrifuged at 100,000 g for 2 h at 4°C to recover the proteoliposomes. To remove filaments, proteins were centrifuged at 12,000 g for 20 min at 4°C before reconstitution and liposomes were then recovered by centrifugation at 12,000 g for 20 min at 4°C. This sample preparation has been used for triplicate error assessment on liposomes of the following composition PC/β-sitosterol/PIP mix 75/15/10 (molar ratio) incubated without protein (Figures S1B,D,F).

### NMR

For ^2^H static ssNMR, we applied a static quadrupolar spin echo sequence (Davis et al., 1976) at the ^2^H frequency of 76.8 MHz on a 500 MHz (11.7 T) Bruker Avance III NMR spectrometer, with a 90° pulse of 3.8 μs, a delay of 40 μs, a recycle delay of 2 s, a spectral window of 500 kHz and a number of scans of 512 at least. We acquired spectra at different temperatures, ranging from 278 to 308 K. Sample temperature was stabilized for 20 min prior to data acquisition. All spectra were processed with TopSpin 4.0.6 (Bruker). An exponential window function with a line broadening factor of 300 Hz was applied prior to Fourier transformation. De-Pake-ing procedure (Bloom et al., 1981; McCabe and Wassail, 1997), first order spectral moments M_1_ and local order parameters |2^*^S_CD_| along the acyl chains of DMPC-d54 were calculated with NMR Depaker (provided by Dr. Sébastien Buchoux) and refined by spectra simulation with NMR-099 (provided by Arnaud Grélard) as described in Beck et al. (2007). Membrane thickness is calculated as described in Grélard et al. (2013). For DMPC-d54/DMPS 90/10 (molar ratio) liposomes, ^2^H static ssNMR was performed on a 300 MHz (7.1 T) Bruker Avance III at 121.50 MHz for 31P with a 90° pulse of 3.3 μs, a delay of 30 μs, a recycle delay of 2 s, a spectral window of 500 kHz and a number of scans of 512 at least. Sample temperature was stabilized for 10 min prior to data acquisition. Error bars on M_1_ and |2^*^S_CD_| are presented in Figure S1 and reflect standard deviations of three independent experiments on the two conditions (see sample preparation 2.2). Error bars for membrane thicknesses are 0.5 Å, based on (Grélard et al., 2013) plus the error contributions from the standard deviations of the |2^*^S_CD_| at each position.

For ^31^P ssNMR, we applied a static Hahn spin echo sequence at the ^31^P frequency of 162 MHz on a 400 MHz (9.4 T) Bruker Avance III HD spectrometer, with a 90° pulse of 8 μs, a delay of 40 μs, a recycle delay of 5 s, a spectral window of 400 ppm and a number of scans of 1,024 at least. Spectra were processed with TopSpin 4.0.6 (Bruker). An exponential window function with a line broadening factor of 200 Hz was applied prior to Fourier transformation.

^31^P magic-angle spinning (MAS) ssNMR was performed on a Bruker Avance III spectrometer with a 4 mm HX probe at the frequency of 121.52 MHz, a MAS rate of 7 kHz and a 2.5 μs 90° pulse-acquisition sequence. Five hundred twelve scans were acquired for both experiments. Pure H_3_PO_4_ was used as external reference.

### Negative Staining Electron Microscopy

Samples were loaded onto previously glow-discharged carbon-coated copper grids and stained with 2% uranyl acetate (w/v) solution. Observations were performed under low-dose conditions on a CM120 120 kV FEI electron microscope using a Gatan USC1000 2k × 2k camera. Clichés were analyzed with the Fiji distribution of ImageJ2 (Schindelin et al., 2012; Rueden et al., 2017).

## Results

### Nanodomain Segregation by RemCA Involves Both PIPs and β-Sitosterol

**Figure 2:**
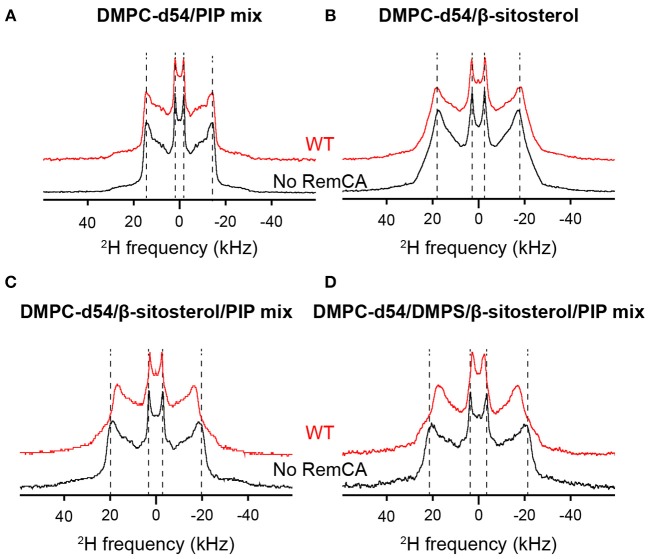
Comparison of ^2^H quadrupolar spin echo spectra acquired at 298K in absence (black) or in presence (red) of RemCA WT. Inner line pairs indicate the Pake doublet frequency of the terminal CD_3_ while outer line pairs indicate the plateau region (usually between positions 2 and 8). Liposome compositions are **(A)** DMPC-d54/PIP mix 90/10, **(B)** DMPC-d54/β-sitosterol 85/15, **(C)** DMPC-d54/β-sitosterol/PIP mix 75/15/10, **(D)** DMPC-d54/DMPS/β-sitosterol/PIP mix 65/10/15/10 (molar ratio), at pH = 7–8.

Using ^2^H ssNMR, we monitored thermotropism, lipid dynamics, and membrane thickness of liposomes containing consecutively the different membrane components that might impact on remorin-driven nanodomain assembly. The lipid systems of choice included, consecutively, perdeuterated phosphatidylcholine (PC, here DMPC-d54), phosphoinositolphosphates (PIPs), β-sitosterol and phosphatidylserine (PS, here DMPS), Figures 1B,C. Deuterium spectra reveal the quadrupolar splittings that can be assigned to the positions of the ^2^H along the acyl chain of the deuterated lipid. The splitting of the so-called Pake doublet depends on the dynamics of the Carbon-Deuterium bond and can be translated into the local order parameters (|2^*^S_CD_|) along the acyl chains of DMPC-d54 by de-Pake-ing (Davis, 1983). To accurately measure the impact of RemCA WT peptides on lipid dynamics we detected the quadrupolar splittings and then computed the order parameters |2^*^S_CD_| in presence and in absence of RemCA. We chose the physiologically relevant temperature of 298K (25°C). Addition of RemCA to liposomes containing PC/PIPs and PC/β-sitosterol did not visually modify the quadrupolar splittings (Figures 2A,B) whereas RemCA has a clear impact on membranes containing PC/β-sitosterol/PIPs and PC/PS/β-sitosterol/PIPs (Figures 2C,D). Membrane-protein interactions generally entail a modification of the lipid dynamic behavior. The selective impact of RemCA points out the importance of β-sitosterol and PIP being simultaneously present to allow for its nanodomain association.

**Figure 3:**
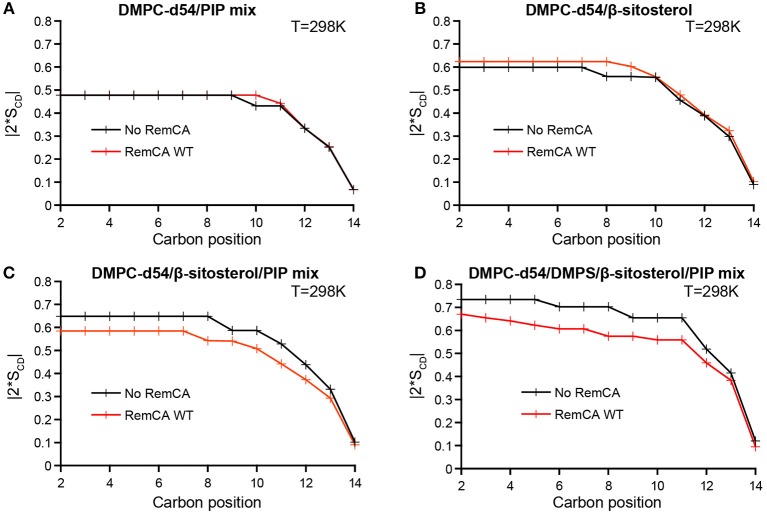
Local order parameters |2*S_CD_| as a function of the carbon positions along the acyl chains of DMPC-d54 in absence (black) or presence (red) of RemCA WT. Liposome compositions are **(A)** DMPC-d54/PIP mix 90/10, **(B)** DMPC-d54/β-sitosterol 85/15, **(C)** DMPC-d54/β-sitosterol/PIP mix 75/15/10, **(D)** DMPC-d54/DMPS/β-sitosterol/PIP mix 65/10/15/10 (molar ratio), at pH = 7–8. Representative error bars are shown in Figure S1C.

Assessing the order parameters along the acyl chain provides a detailed view on the impact of RemCA on the membrane dynamics (Figure 3). No significant change in |2^*^S_CD_| was observed in PC/PIPs and PC/β-sitosterol liposomes upon addition of RemCA (Figures 3A,B), despite a slight increase of the order parameter in the presence of β-sitosterol. PC/β-sitosterol/PIPs and PC/PS/β-sitosterol/PIPs liposomes display lower |2^*^S_CD_| along their acyl chains in the presence of RemCA, indicating a PIP- and sterol-dependent interaction between RemCA and the membrane (Figures 3C,D). The chosen lipids display a consecutively rigidifying effect on the membrane (Figure S2). RemCA then increases the lipid mobility in the membranes of the complex lipid mixtures PC/β-sitosterol/PIPs and PC/PS/β-sitosterol/PIPs. Addition of PS does not significantly enhance the observed effect of RemCA on liposomes containing β-sitosterol/PIPs (Figure 3D). To monitor whether the impact of the peptide depends exclusively on the simultaneous presence of PIPs and β-sitosterol, we tested its effect also on PC/PS-containing liposomes and we observe no detectable change of the |2^*^S_CD_| (Figure S3). The representative error for this type of sample preparation (Figure S1C) is below 0.9% at carbon positions 2–6.

**Figure 4:**
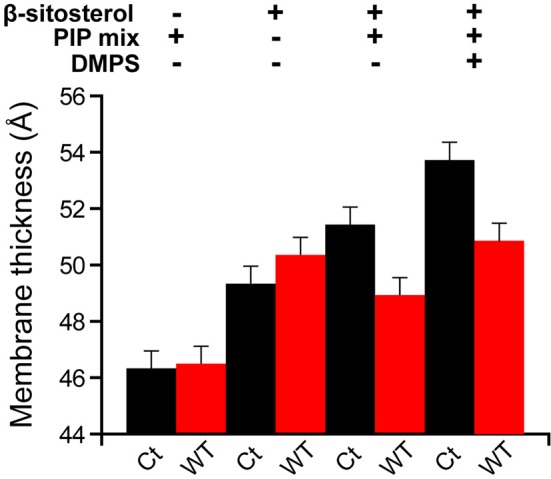
Membrane thickness at 298K calculated from the corresponding |2*S_CD_| (Figure 2) in absence (black) or presence (red) of RemCA WT. Liposome compositions are, from left to right, DMPC-d54/PIP mix 90/10, DMPC-d54/β-sitosterol 85/15, DMPC-d54/β-sitosterol/PIP mix 75/15/10 and DMPC-d54/DMPS/β-sitosterol/PIP mix 65/10/15/10 (molar ratio), at pH = 7–8. Error bars are assessed as described in Figure S1E.

The local order parameter |2^*^S_CD_| can further be translated into the average membrane thickness (Grélard et al., 2013) at 298K (Figure 4). PC/PIPs and PC/β-sitosterol liposomes without and with RemCA display similar membrane thicknesses (46.3 Å vs. 46.5 Å and 49.3 Å vs. 50.4 Å) whereas the membrane thickness of PC/β-sitosterol/PIPs and PC/PS/β-sitosterol/PIPs decreases slightly in the presence of RemCA (51.4 Å−48.9 Å and 52.7 Å−50.9 Å, respectively).

The first order spectral moments M_1_, which can be extracted from the deuterium spectra, report on the bilayer phase behavior and thermotropism (Figure S4). The inflection point between higher M_1_ values indicating a gel phase to lower ones pointing to a fluid phase reports on the phase transition. For all lipid compositions, the phase transition temperatures (Tm) seem to remain close to the Tm of DMPC-d54 alone, 293K, but no clear phase transitions were detected in the presence of β-sitosterol. We observe a clear thermotropic transition in the absence of sterols, but only a slow descent of M_1_ in their presence. Sterols might broaden the primary transition or it might be below the chosen temperature range (Dufourc, 2008). The presence of RemCA WT peptides does not have a significant effect on M_1_ values nor on Tm across the range of temperature considered, 278–308 K. ^31^P NMR confirms the presence of lamellar phases in liposomes in all samples, as well as small vesicles in presence of PIPs (Figure S5).

Taken together with the |2^*^S_CD_| analysis, our results show that upon interaction with membranes of relevant lipid compositions (PC/β-sitosterol/PIP and PC/PS/β-sitosterol/PIP), RemCA WT binds to the lipid bilayer without disrupting its structural integrity and lipid phase, while exerting a noticeable effect on its internal dynamics, reflected by the variations of the order parameters.

### pH Dependency of Protein-Lipid Interactions in Nanodomain Association

**Figure 5:**
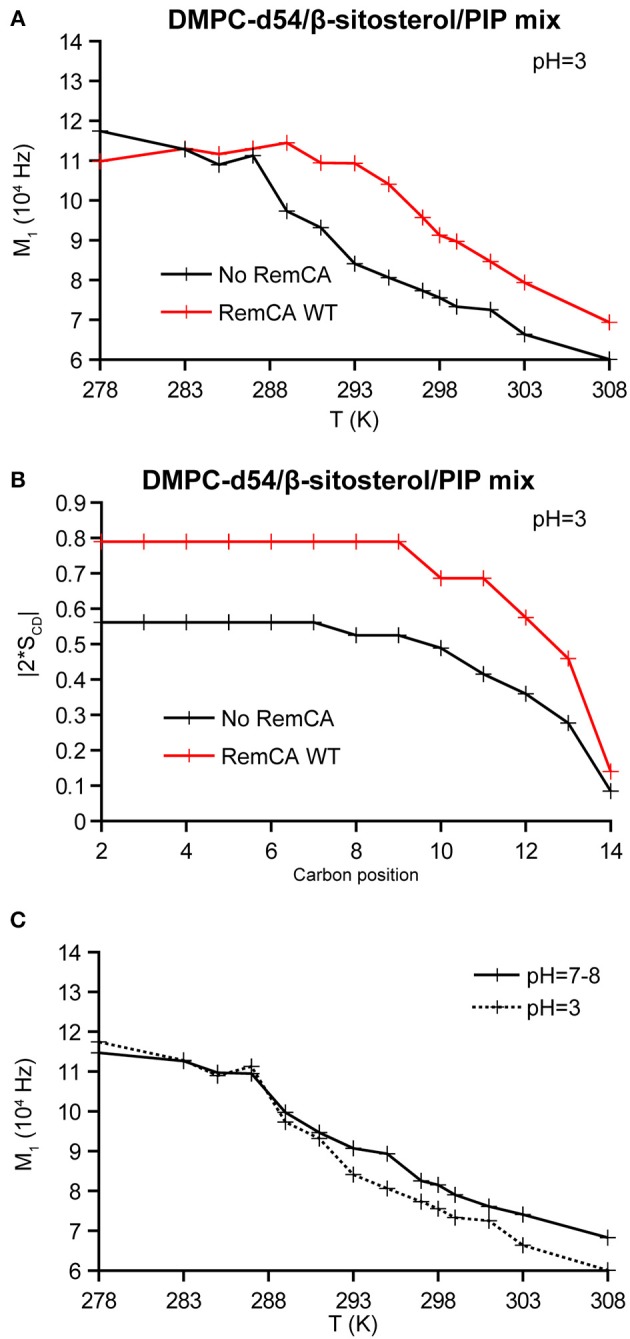
**(A)** M_1_ as a function of the temperature for liposomes containing PIPs, hydrated with 1% acetate pH = 3 in absence (black) or in presence (red) of RemCA WT at peptide/lipid ratio of 1/25. **(B)** Local order parameters |2*S_CD_| as a function of the carbon position along the acyl chains of DMPC-d54 in presence of RemCA WT at pH = 3–4. **(C)** M_1_ as a function of the temperature for liposomes, in 1% acetate pH = 3 (dotted lines) or with 260 mM Tris pH = 8 (full lines). Liposome composition is: DMPC-d54/β-sitosterol/PIP mix 75/15/10 (molar ratio). Representative error bars are shown in Figures S1A,C.

At low pH (pH≈3–4), polyphosphate PIPs lose parts of their negative charges present at pH = 7–8 (Kooijman et al., 2009). Though, to our knowledge, no values on the pH dependency of the charges for all PIPs of the PIP mix have been documented, in analogy to PI(3,5)P_2_, the PIPs of the PIP mix (including PI4P) should be neutralized at pH≈3–4. Moreover (Redfern and Gericke, 2004) showed that di-palmitoyl phosphoinositol mono-phosphates were able to segregate into phosphatidylinositol-enriched microdomains at pH = 7–9.5 but not at pH = 4. To obtain insights into the role of electrostatic interactions during the RemCA-membrane interplay, we decided to test how the pH value influences the impact of RemCA on nanodomain-mimicking membranes. To assess the lipid interactions during RemCA-membrane association, we co-solubilised synthetic RemCA WT peptides in various liposome preparations (%/% molar ratio): DMPC/PIP mix 90/10, DMPC/β-sitosterol 85/15, DMPC/β-sitosterol/PIP mix 75/15/10 and DMPC/DMPS/β-sitosterol/PIP mix 65/10/15/10. Peptides used in our study carried impurities (<10% w/w), such as TFA, which acidified the samples to pH = 3–4 during peptide-containing liposome preparation, as judged by pH paper. We therefore chose to buffer our samples to an approximate pH = 7–8, as judged by pH paper. To reveal the RemCA/membrane interactions at low pH, we also rehydrated our samples in pure 1% acetate pH = 3 during proteoliposome reconstitution (final pH = 3). Monitoring phase behavior (Figure 5A), spectral line shapes (Figure S6), and local order parameters |2^*^S_CD_| (Figure 5B) at low pH revealed the significant effect of the head group charges on RemCA-lipid interactions. RemCA, when in contact with low pH nanodomain-mimicking membranes has the inverse effect on the phase behavior, as observed by an increase of the first spectral moment M1 (Figure 5A), and local order parameter (Figure 5B) as compared to neutral pH (Figure 3C and Figure S4C). Thus, the impact of the peptide on membrane dynamics is significantly modified because of the lower pH. ^31^P MAS ssNMR revealed significant chemical shift perturbations as a function of pH (Figure S7). Based on (Kishore and Prestegard, 2003; Müller et al., 2004), we tentatively assigned the four visible signals, from higher to lower chemical shifts: 4-phosphate (4-P)-, 4,5-bisphosphate (4,5-P_2_)-monoester, PS and PC. The diester groups might be obscured by the PS and PC signal. Assignment of 4,5-P_2_- remained ambiguous as a second peak was expected. Signals assigned to the monoesters witnessed a heavy shift between spectra at pH = 7–8 and revealing important modifications of their chemical environment which may be attributed to a change in their protonation state (van Paridon et al., 1986).

The acidic pH entails a negligible effect on phase behavior (Figure 5C) whereas it significantly modifies the local order parameter at 298K (black curves, Figure 3C vs. Figure 5B).

### K192 and K193 Are Critical Residues in the Targeting of PIPs by RemCA WT

**Figure 6:**
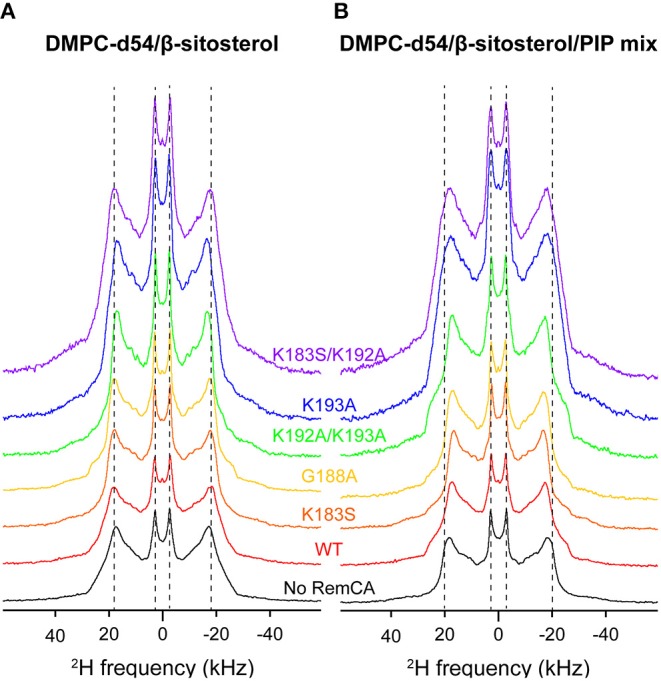
Comparison of ^2^H quadrupolar spin echo spectra acquired at 298K in presence of RemCA WT and mutated RemCA. Inner line pairs indicate the Pake doublet frequency of the terminal CD_3_ while the outer line pairs indicate the plateau region (usually between positions 2 and 8). Liposome compositions are **(A)** DMPC-d54/β-sitosterol 85/15 and **(B)** DMPC-d54/β-sitosterol/PIP mix 75/15/10 (molar ratio), at pH = 7–8. Above RemCA WT, peptides with a similar effect on membranes in presence of PIP mix (K183S, G188A, K192A/K193A) followed by those with no discernible effect (K193A, K183A/K192A).

**Figure 7:**
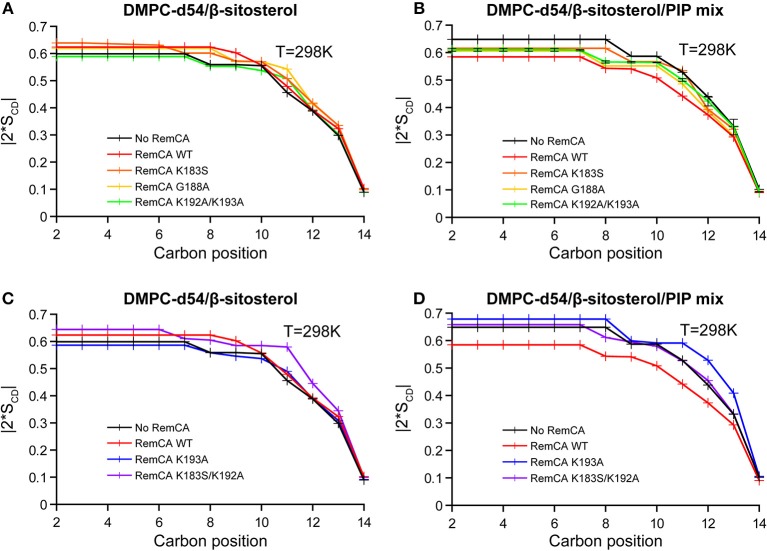
Local order parameters |2*S_CD_| as a function of carbon positions along the acyl chains of DMPC-d54 in presence of RemCA WT or mutated RemCA. Liposome compositions are **(A,C)** DMPC-d54/β-sitosterol 85/15 and **(B,D)** DMPC-d54/β-sitosterol/PIP mix 75/15/10 (molar ratio), at pH = 7–8. Representative error bars are shown in Figure S1C.

Based on a pre-established list of mutants impaired for both PM and nanodomain targeting (Gronnier et al., 2017), we employed mutated RemCA peptides to evidence the concerted role of lipid-amino acid interactions involving the specific targeting of PIPs. According to previous molecular dynamics experiments, residues K192 and K193 play a key role in the targeting of PI4P by *St*REM1.3. We limited ourselves to five mutants (Gronnier et al., 2017): G188A, a negative control that should show no specific difference to the wild-type peptide; K183S, K193A, K183S/K192A, and K192A/K193A, localizing less efficiently into nanodomains. To trace the influence of each mutation on the peptide-PIP interaction, each mutant and the WT were co-solubilised in liposomes of two different lipid compositions: PC/β-sitosterol 85/15 and PC/β-sitosterol/PIP mix 75/15/10 (molar ratio). We then determined the local order parameter |2^*^S_CD_| of DMPC-d54 at 298K for every sample (WT, K183S, G188A, K192A/K193A, K193A, and K183S/K192A) (Figures 6, 7). In absence of PIPs, the effect of the peptides on the quadrupolar splitting and the local order parameter |2^*^S_CD_| of the liposomes does not differ between RemCA WT and the mutants (Figures 6A, 7A,C); the impact of RemCA WT and the mutants is undetectable or entails a slight increase of the local |2^*^S_CD_|. In presence of PIPs, we observe two different behaviors (Figures 6B, 7B,D). The wild type peptide (RemCA WT) and a group of mutants (K183S, G188A, and K192A/K193A) have a pronounced effect on the membranes, decreasing |2^*^S_CD_| all along the acyl chains of DMPC-d54. Importantly, this effect is abolished for a second group composed of the mutants K193A and K183S/K192A (Figures 6B, 7B,D), exposing an undetectable or a slightly increasing effect on the |2^*^S_CD_|. This observation suggests that mutants from the first group, together with RemCA WT, interact with PIP-containing liposomes whereas peptides from the second group do not (Figures 6, 7). Unexpectedly, the double mutant K192A/K193A still binds to liposomes containing PIPs, possibly through an alternative mechanism involving K183. The representative error for this type of sample preparation (Figure S1C) is below 0.9% at carbon positions 2–6.

**Figure 8:**
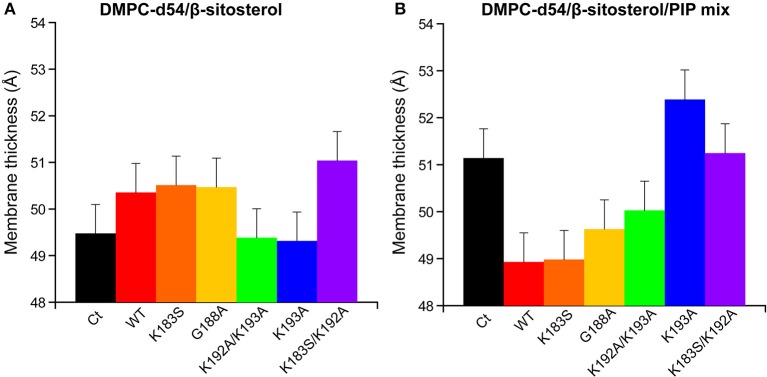
Membrane thickness at 298K calculated from the corresponding |2*S_CD_| (Figure 6). Liposome compositions are **(A)** DMPC-d54/β-sitosterol 85/15 and **(B)** DMPC-d54/β-sitosterol/PIP mix 75/15/10 (molar ratio), at pH = 7–8. Error bars are assessed as described in Figure S1E.

Measurements of the membrane thickness reflect a comparable trend, as expected, since they rely on the ^2^H NMR data. The membrane thicknesses without PIPs are all within the same range of ≈50 Å. Liposomes containing PIPs are split in the same two groups as described above with RemCA WT, K183S, G188A, or K192A/K193A decreasing membrane thickness more or less significantly (≈49 Å) whereas RemCA K193A or K183S/K193A have no effect (≈51 Å) (Figure 8). Our data corroborate the *in vivo* results that K192 and K193 are critical residues in the targeting of PIPs by RemCA. However, the results reveal that the *in vivo* membrane association of *St*REM1.3 relies on a more complex behavior of the positively charged residues during the trimerised protein-membrane interaction.

Monitoring M_1_ as a function of temperature, we detect no important impact of the different mutants and the WT on the phase behavior (Figure S8). Above 293K, M_1_ values in presence of PIP mix seem to spread out a little more than without PIP mix. All samples contain large liposomes in lamellar phase along with smaller vesicles in presence of PIP mix (Figure S9). Our data point to different effects of the different mutations, larger than the error margins for repetition of the identical conditions. However, considering possible uncertainties introduced by other sources (e.g., varying impurities in different peptide stocks) future interpretation of these results should be handled with care.

### Oligomerisation Modifies RemCA Behavior on Nanodomain-Mimicking Membranes

We have previously demonstrated (Martinez et al., 2018) that disrupting the coiled-coil region of *St*REM1.3 would partially disrupt membrane targeting. While we hypothesize that the intrinsically disordered domain, responsible for protein-protein interactions, and the coiled-coil homo-oligomerisation domain do not directly interact with the nanodomains, they could indirectly influence nanodomain targeting. To monitor the effect of the two domains, we designed two protein constructs for NMR investigation. We expressed and purified the truncated *St*REM1.3 (REM_86−198_), bearing only RemCA and the coiled-coil domain (Martinez et al., 2018), and the full length *St*REM1.3 including also the IDD.

We achieved setting up a protocol to produce high quantities of *St*REM1.3 in *E.coli* (about 20 mg/L of culture medium) (Figures S10, S11A). Both protein constructs, REM_86−198_ and *St*REM1.3, were purified to high purity (Figure S11B). We avoided reconstitution into liposomes by co-solubilisation to mitigate the risk of misfolding and non-native interactions with lipids. Instead, we incubated the proteins with preformed liposomes overnight at 30°C, using two lipid compositions: DMPC-d54/β-sitosterol 85/15 or DMPC-d54/β-sitosterol/PIP mix 75/15/10 (molar ratio). For each proteoliposome sample, about 40–50% of the proteins were pelleted by ultracentrifugation, as could be assessed by absorbance at 280 nm of the supernatant fractions and SDS-PAGE of the pellets (Figure S12). We used deuterium NMR to probe the lipid order in presence of the two remorin domains.

**Figure 9:**
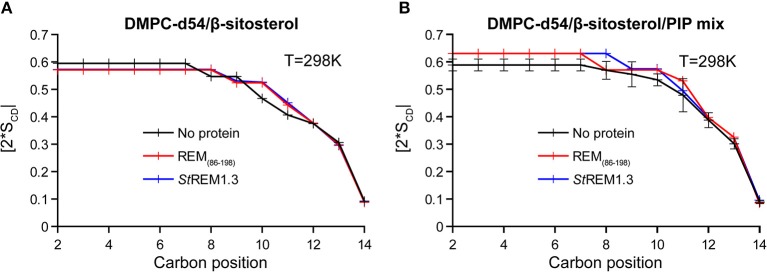
Local order parameters |2*S_CD_| as function of the carbon position along the acyl chains of DMPC-d54 in presence of REM_86−198_ or *St*REM1.3. Liposome compositions are **(A)** DMPC-d54/β-sitosterol 85/15 and **(B)** DMPC-d54/β-sitosterol/PIP mix 75/15/10 (molar ratio), at pH = 7–8. Representative error bars are shown in Figure S1D.

**Figure 10:**
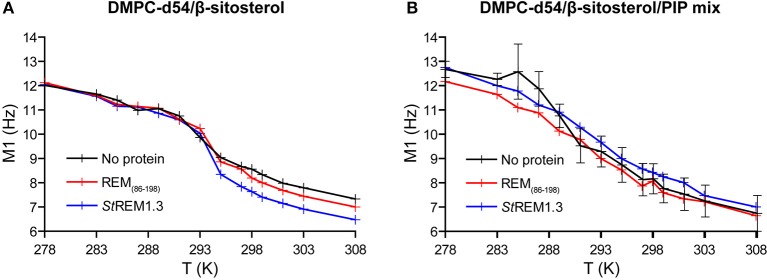
Variation of first order spectral moments M_1_ as a function of temperature T in presence of REM_86−198_ or *St*REM1.3. Liposome compositions are **(A)** DMPC-d54/β-sitosterol 85/15 and **(B)** DMPC-d54/β-sitosterol/PIP mix 75/15/10 (molar ratio). Error bars are standard deviations of three independent experiments, as shown in Figure S1B.

Local order parameters |2^*^S_CD_| of PC-d54/β-sitosterol membranes are similar whether in absence or presence of both REM_86−198_ and *St*REM1.3, no protein-membrane interactions seem to occur (Figure 9A and Figure S13A). In contrast, DMPC-d54/β-sitosterol/PIP liposomes containing REM_86−198_ or *St*REM1.3 witnessed a similar increase of the order parameter |2^*^S_CD_| all along their acyl chains (Figure 9B and Figure S13B). In accordance, membrane thicknesses were similar in absence of PIPs but increased comparably in presence of PIPs and one of the proteins (Figure S14). The representative error for this type of sample preparation (Figure S1D) is below 3.7% at carbon positions 2–7. The samples contain large liposomes in lamellar phase with a small isotropic peak in presence of PIPs (Figure S15). All samples displayed a Tm around 293K (Figure 10, representative error in Figure S1B). When compared to the monomeric anchor RemCA, REM_86−198_ and *St*REM1.3 show an inverse effect on nanodomain-mimicking membranes, revealing the crucial impact of remorin oligomerisation during nanodomain clustering.

In contrast, both constructs, REM_86−198_ and *St*REM1.3, display a very similar behavior on the membranes, indicating that the non-phosphorylated intrinsically disordered domain does not influence remorin nanodomain targeting. The sample preparation used for measuring the effects of the two longer constructs should lead to reduced detectable readout range since predominantly the outer leaflet of the liposomes will be accessible. In line with this view, our results indeed show smaller effects for all tested conditions. Consequently, the data and interpretation should be handled with care.

### Transient Higher-Order Oligomer Formation Could Stabilize Nanodomain Clustering

When incorporating the constructs REM_86−198_ and *St*REM1.3 into liposomes, negative staining electron microscopy revealed the presence of protein filaments (Figure S16). Notably, this observation mostly concerned samples without PIPs (Figures S16A,B,E,F) whereas in the presence of PIPs very few filaments were observed (Figures S16C,D,G,H). Since there is no significant effect of either protein on liposomes in absence of PIPs and filament formation is significantly reduced in the presence of PIPs, we assume that the filaments do not directly interfere with liposomes. Nevertheless, we attempted to remove filaments by pelleting at lower centrifugal force before reconstitution. Removing the filamentous objects before reconstitution into liposomes caused both proteins to remain mostly in solution when exposed to the preformed liposomes, as assessed by absorbance at 280 nm (2.5 and 1.7 in solution before reconstitution, 2.8 and 1.7 in supernatant after reconstitution and liposome pelleting for REM_86−198_ and *St*REM1.3, respectively). Absorbance measurement may be biased by remaining small liposomes. However, few proteins were still incorporated into the liposomes, for REM_86−198_ only in the presence of PIPs, as the band seems to be absent, even though it might be hidden under the liposome smear (Figure S17). No filaments were found on the liposomes (example given for REM_86−198_, Figure S18). We could not detect an effect of this low protein amount on the membrane dynamics as reflected in the local order parameter |2^*^S_CD_| of DMPC-d54 (Figure S19). Since filaments do not seem to have a direct impact on liposomes and their formation is reduced in the presence of PIPs, transient higher-order oligomer formation might stabilize protein-lipid interactions during membrane association.

## Discussion

Our results shed light on the detailed mechanisms of *St*REM1.3-driven nanodomain clustering. We dissect the roles of the three *St*REM1.3 domains, the C-terminal anchor RemCA, the coiled-coil oligomerisation domain and the intrinsically disordered domain (IDD), implicated in protein-protein interactions.

We have monitored a fluidifying effect of RemCA on the membrane, assessed by the decrease of the local carbon-deuterium order parameter all along the acyl chains of DMPC-d54, only in presence of PIPs and β-sitosterol simultaneously. RemCA's membrane interactions therefore depend on the presence of both PIPs and β-sitosterol during nanodomains clustering, as we have suggested earlier (Raffaele et al., 2009; Gronnier et al., 2017). Moreover, addition of DMPS, another negatively charged phospholipid, does not modify this behavior, corroborating RemCA's genuine specificity for PIP and β-sitosterol.

RemCA peptides lacking K192 or K193, namely RemCA K183S/K192A or K193A, do not show any fluidifying effect on nanodomain-mimicking membranes, highlighting the important role of these two positively charged residues. In the absence of PIPs RemCA and several mutants show a slight increase of the lipid order parameters in the presence of β-sitosterol, which could be attributed to a low overall membrane binding without the formation of lipid clusters. K183S and K192A/K193A mutants still interact with the nanodomain-mimicking membranes such as the wild-type RemCA. These two mutants behave similarly to K183S/K192A or K193A, when considering their segregation into nanodomains in *St*REM1.3 *in vivo*, detected by Single-Particle Tracking Photoactivated Localization Microscopy, spt-PALM (Gronnier et al., 2017). Their behavior on nanodomain-mimicking membranes *in vitro* therefore suggests a more complex interplay between the different positive charges in the intact *St*REM1.3 *in vivo* (Gronnier et al., 2017), possibly influenced by the oligomerisation and the subsequent trimeric exhibition of the anchor RemCA. However, our results are coherent with specific electrostatic interactions between K192 and K193 and the polar head of PI4P from the PM's inner leaflet (Gronnier et al., 2017).

In the K192A/K193A mutant, still displaying specificity for PIPs, the replacement of two consecutive positively charged lysines by two consecutive hydrophobic alanines might allow an alternative folding and binding mechanism. However, we cannot exclude the possibility of errors that might not have been monitored by our error assessment, so we suggest treating the data with care.

Our findings show that negative PIP head group charges should foster RemCA nanodomain-mimicking membrane association. We therefore tested the impact of the pH on RemCA-membrane interactions. The impact of RemCA on phase behavior and acyl chain mobility at acidic pH is inversed compared to neutral pH, i.e., RemCA reduces acyl chain mobility in the nanodomain-mimicking membrane in acidic conditions. This might rely on an alternative binding mode of RemCA to the lipid bilayer, potentially reflecting an unspecific overall membrane binding, as is also the case for low binding of the peptide and its mutants in the presence of β-sitosterol. The electrostatic PIP-RemCA interactions present at physiological conditions should therefore represent a crucial actor during remorin nanodomain clustering.

We then examined the membrane- and nanodomain-association of the C-terminal anchor in conjunction with the coiled-coil oligomerisation domain REM_86−198_ and the full-length *St*REM1.3, including the intrinsically disordered region. In line with our knowledge on *St*REM1.3, they only associate to nanodomain-mimicking membranes, i.e., containing PIPs and β-sitosterol responsible for *St*REM1.3 nanodomain segregation. Their effect on the membrane dynamics is very similar, indicating that the intrinsically disordered domain does not directly influence the nanodomain association. However, use of phosphodead *St*REM1.3 S74A/T86A/S91A and phosphomimetic *St*REM1.3 S74D/T86D/S91D mutants revealed a phosphorylation-dependent change in nanodomain organization *in vivo* (Perraki et al., 2018), implying the *St*REM1.3's intrinsically disordered region indirectly modifies the membrane-associating behavior, possibly by altering the interactions with other proteins or itself.

**Figure 11:**
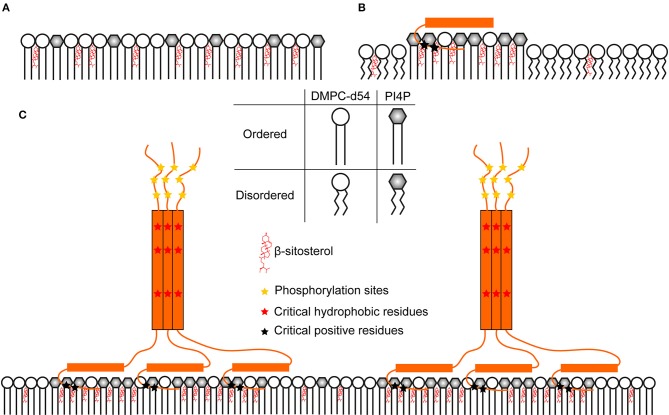
Hypothetic model of a comprehensive nanodomain clustering mechanism consolidating the structural data on *St*REM1.3. **(A)** PIP mix and sterols increase lipid order over the whole membrane because of their homogeneous distribution **(B)** RemCA peptides could bind and cluster PIP altogether and recruit sterols in their vicinity. This would reduce the presence of sterols and PIPs in DMPC-d54-rich regions, lowering the |2*S_CD_| of the latter. **(C)** *St*REM1.3 forms homo-trimers, bringing RemCA domains in vicinity. This might increase the rigidifying effect more efficiently over the whole membrane, considering the size and the stability of the associated objects.

Both REM_86−198_ and the full-length *St*REM1.3 have an inverse effect on nanodomain-mimicking membrane dynamics when compared to RemCA WT. The anchoring domain alone is in a monomeric state and therefore represents the lipid specificity of the peptide alone (Figures 11A,B), presumably fostering the formation of lipid clusters. In the absence of RemCA, the lipid dynamics could represent the homogeneous rigidifying effect of intercalated PIP/sitosterol (Figure S1) along the reporter DMPC acyl chain (Figure 11A). When RemCA is present, it might cluster the PIP/sitosterol moieties and therefore have a fluidifying effect on the reporter DMPC acyl chains (Figure 11B). Including the coiled-coil domain drastically increases the complexity of the native trimeric *St*REM1.3 structure, containing three RemCA anchors. REM_86−198_ and *St*REM1.3 would reflect the rigidifying impact of the clustered protein on the nanodomain-mimicking membranes (Figure 11C) with the inner bilayers of the liposomes unaffected and the outer exposed to the trimeric remorin. The protein and lipid clustering might reflect driving mechanisms of nanodomain formation in native membranes. Again, we cannot exclude the possibility of errors that might not have been monitored by our error assessment, in consequence we suggest treating the data with care.

We found that REM_86−198_ and *St*REM1.3 formed filaments even at low protein concentration, when we proceeded to liposome reconstitution (Figure S16). Filament formation observed by EM was much reduced by the presence of PIP (Figures S16C,D,G,H) and the pelleted liposomes contained a significantly higher amount of protein in the presence of PIP (Figure S12), indicating that the protein is incorporated into membranes. When removing filaments by moderate centrifugation, the relative amount of proteins incorporated into liposomes drastically decreases, indicating that the presence of transient higher-order oligomers might favor the incorporation of trimeric remorins into the nanodomain-mimicking membranes. Although filaments are unlikely to be stable *in vivo*, the underlying ability of *St*REM1.3 to cluster with its peers into higher-order complexes could be biologically relevant. In line with this results, disrupting the coiled-coil region resulted in a partial loss of membrane targeting, indicating this domain is required to ensure a tight binding to the PM (Martinez et al., 2018).

## Conclusion

Gaining mechanistic insights into the interactions and protein/membrane structures governing nanodomain formation remains difficult because of the complexity of the native protein-bilayer system. Here, we employed a ^2^H ssNMR-based methodology to tackle the lipid-protein interactions and mechanisms behind nanodomain clustering driven by *St*REM1.3. Using a divide-and-conquer approach, we deciphered the roles of the membrane anchor RemCA in isolation, REM_86−198_, including the coiled-coil domain responsible for trimerisation and the intact *St*REM1.3. We seek to uncover the essential electrostatic interactions between RemCA's positive residues and the negatively charged PIP head groups involved in nanodomain formation. Our data moreover suggest that nanodomain clustering depends on the evolutionary evolved trimeric structure, which can only be partially represented by the monomeric anchor. The trimers of remorins appear to confer nanodomain clustering while the pure protein-lipid association relies mostly on the membrane anchor RemCA. Furthermore, we reveal that transient higher-order oligomer formation might stabilize the *in vivo* association of remorins to nanodomains, which is further supported by the structural conservation of the oligomerisation domain in the six phylogenetic groups (Raffaele et al., 2007).

Our results shed new light on the essential role of certain specific electrostatic protein-lipid interactions and protein oligomerisation properties toward understanding the driving forces in the nanoclustering of *St*REM1.3. We propose a more general picture of the relevance of oligomerisation, a character often present in nanodomain-segregating proteins. To gain more precise notions on the mechanisms behind lipid domain assembly, it will be vital to understand the structural implications of protein oligomerisation and the lipid-protein interactions from a protein structural point of view.

## Data Availability Statement

All datasets generated for this study are included in the manuscript/Supplementary Files.

## Author Contributions

ALe, DM, AG, MB, AT, and EM performed the experiments. ALe, DM, ALo, SM, and BH interpreted the data. ALo, SM, and BH designed the experiments. BH conceptualized the research. All authors revised and edited the manuscript.

### Conflict of Interest

The authors declare that the research was conducted in the absence of any commercial or financial relationships that could be construed as a potential conflict of interest.
