# Supplementary material for: Nanodomain Clustering of the Plant Protein Remorin by Solid-State NMR
Source: Front Mol Biosci. 2019 Oct 15;6:107. doi: 10.3389/fmolb.2019.00107 (PMC6803476; doi:10.3389/fmolb.2019.00107)
Supplement: Supplementary file 1 [file Data_Sheet_1.PDF]

Supplemental information

Nanodomain clustering of the plant protein remorin by solid-state NMR

Anthony Legrand[1,2], Denis Martinez[1], Axelle Grélard[1], Melanie Berbon [1], Estelle Morvan[3], Arpita Tawani[1], Antoine Loquet[1], Sébastien Mongrand[2], Birgit Habenstein[1*]

[1] Institute of Chemistry & Biology of Membranes & Nanoobjects (UMR5248 CBMN), IECB, CNRS, Université Bordeaux, Institut Polytechnique Bordeaux, All. Geoffroy Saint-Hilaire, 33600 Pessac, France

[2] Laboratoire de Biogenèse Membranaire – UMR 5200 – CNRS, Université de Bordeaux, 71 Avenue Edouard Bourlaux, 33883 Villenave d'Ornon Cédex, France

[3] European Institute of Chemistry and Biology – UMS3033/US001 – 2 Rue Robert Escarpit, 33607 Pessac, France

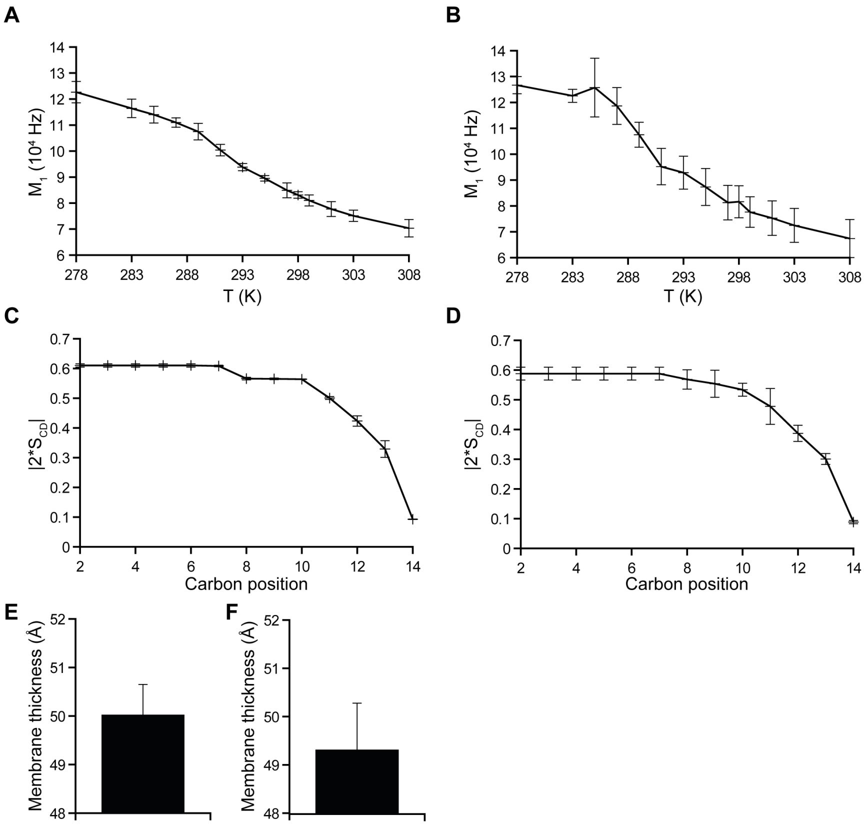

Figure S1. Estimation of the experimental errors for the peptide co-reconstitution method (A, C, E) or liposome preparation for protein constructs (B, D, F) for M_1 (A, B), $|2*S_{CD}|$ (C, D) and membrane thickness assessment (E, F). Error bars are the standard deviations of three independent experiments, as described in section 2.2.

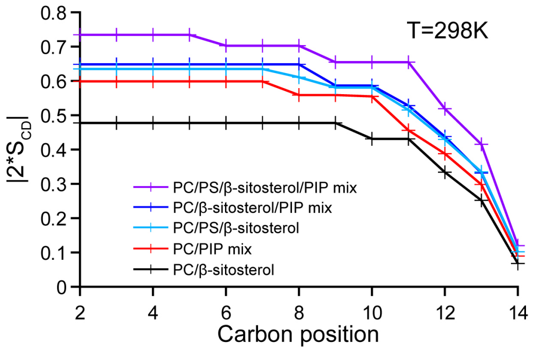

Figure S2. Comparison of the local order parameters $|2*S_{CD}|$ as a function of the carbon position along the acyl chains of DMPC-d54 for the four lipid systems of this study, at 298K. Liposome compositions are: DMPC-d54/β-sitosterol 85/15, DMPC-d54/PIP mix 90/10, DMPC-d54/β-sitosterol/PIP mix 75/15/10, DMPC-d54/DMPS/β-sitosterol/PIP mix 65/10/15/10 (molar ratio) at pH=7-8. Representative error bars are shown in **Figure S1C**.

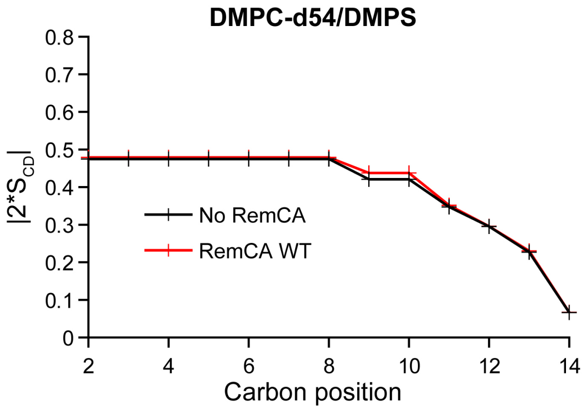

Figure S3. Local order parameters $|2*S_{CD}|$ as a function of the carbon positions along the acyl chains of DMPC-d54 in absence (black) or presence (red) of RemCA WT. Liposome composition is DMPC-d54/DMPS 90/10 (molar ratio), at pH=7-8. Representative error bars are shown in **Figure S1C**.

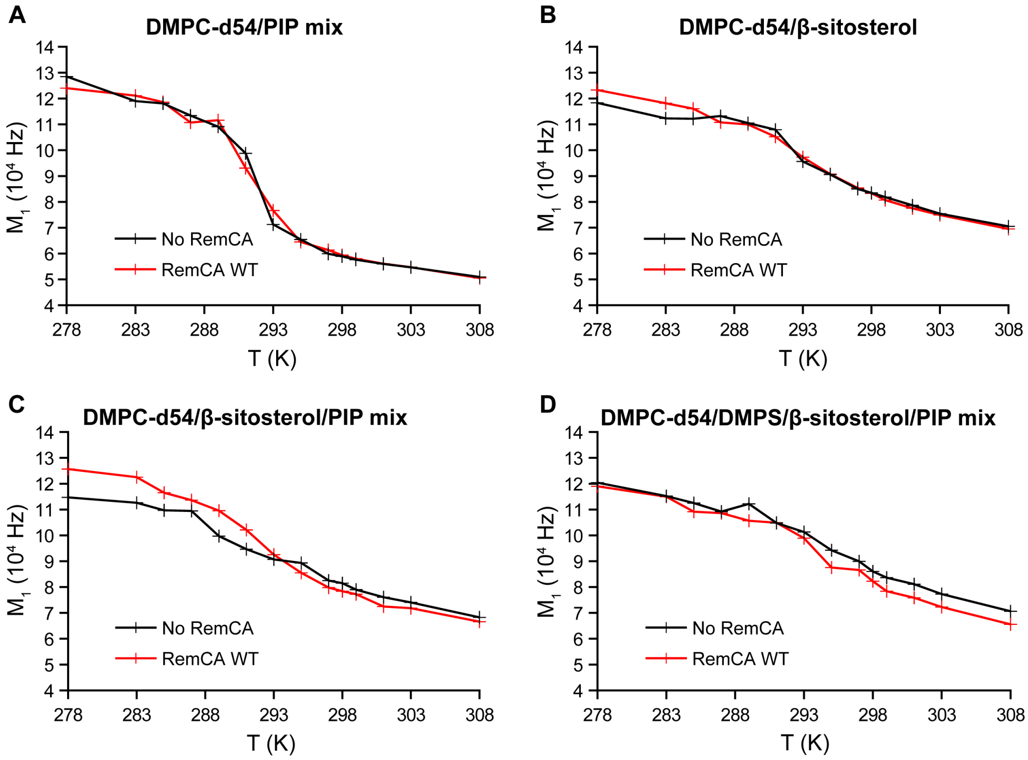

Figure S4. Variation of the first order spectral moments M_1 as a function of the temperature T in absence (black) or presence (red) of RemCA WT. Liposome compositions are (A) DMPC-d54/PIP mix 90/10, (B) DMPC-d54/β-sitosterol 85/15, (C) DMPC-d54/β-sitosterol/PIP mix 75/15/10, (D) DMPC-d54/DMPS/β-sitosterol/PIP mix 65/10/15/10 (molar ratio), at pH=7-8. Representative error bars are shown in **Figure S1A**.

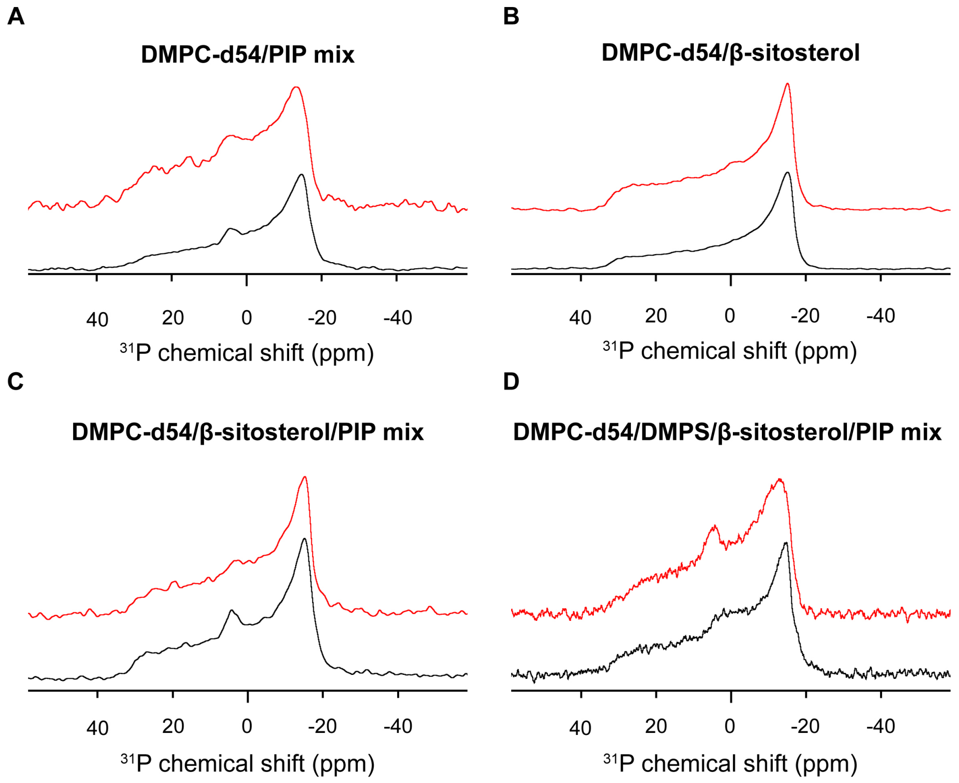

Figure S5. ^{31}P Hahn echo spectra in absence (black) or presence (red) of RemCA WT for different lipid systems. Liposome compositions are (A) DMPC-d54/PIP mix 90/10, (B) DMPC-d54/β-sitosterol 85/15, (C) DMPC-d54/β-sitosterol/PIP mix 75/15/10, (D) DMPC-d54/DMPS/β-sitosterol/PIP mix 65/10/15/10 (molar ratio), at pH=7-8

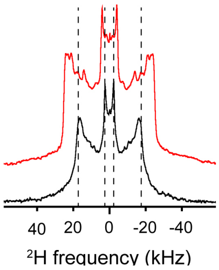

Figure S6. Comparison of ^2H quadrupolar spin echo spectra acquired at 298K in absence (black) or in presence (red) of RemCA WT in liposomes containing PIPs, hydrated with 1% acetate pH=3. The inner line pair indicates the Pake doublet frequency of the terminal CD_3 while the outer line pair indicates the plateau region (usually between positions 2 and 8). Liposome composition is: DMPC-d54/βb-sitosterol/PIP mix 75/15/10 (molar ratio), at pH=3

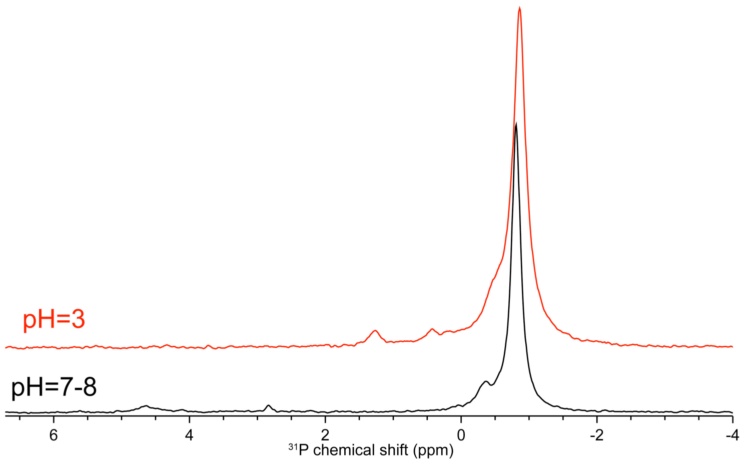

Figure S7. ^{31}P MAS ssNMR on DMPC-d54/β-sitosterol/PIP mix 75/15/10 (molar ratio) in presence of RemCA WT at pH=7-8 (black) or pH=3 (red).

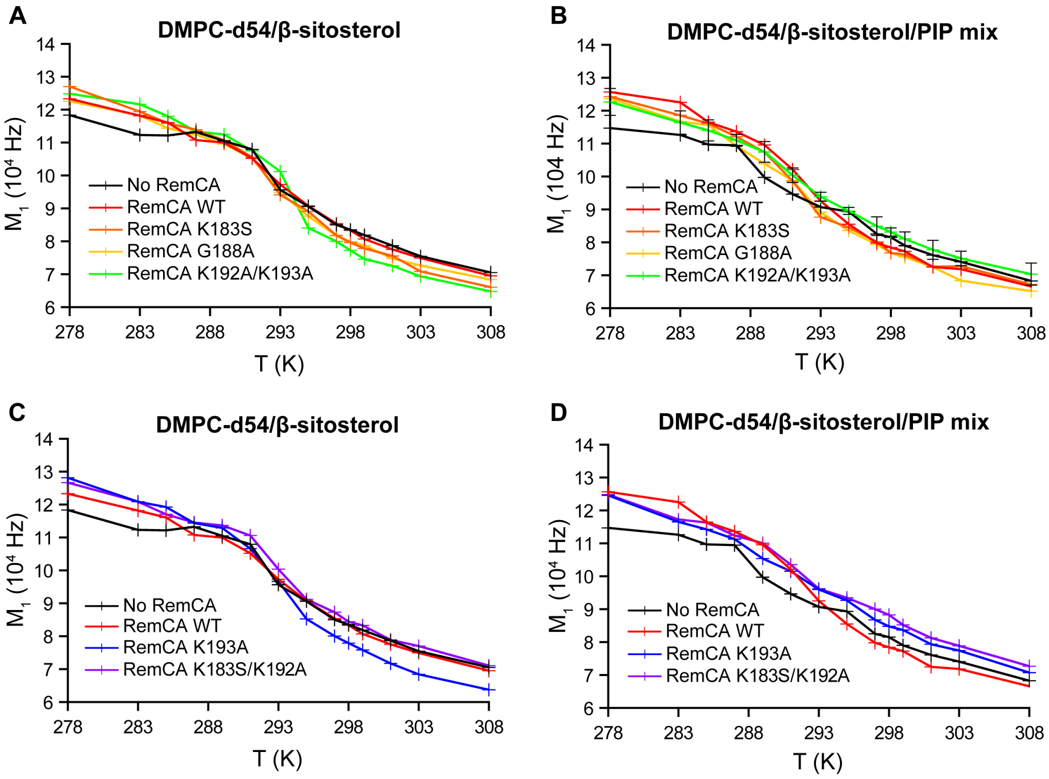

Figure S8. Variation of first order spectral moments M_1 as a function of the temperature T in presence of RemCA WT or mutated RemCA. Liposome compositions are (A, C) DMPC-d54/β-sitosterol 85/15, (B, D) DMPC-d54/β-sitosterol/PIP mix 75/15/10 (molar ratio), at pH=7-8. Representative error bars are shown in **Figure S1A.**

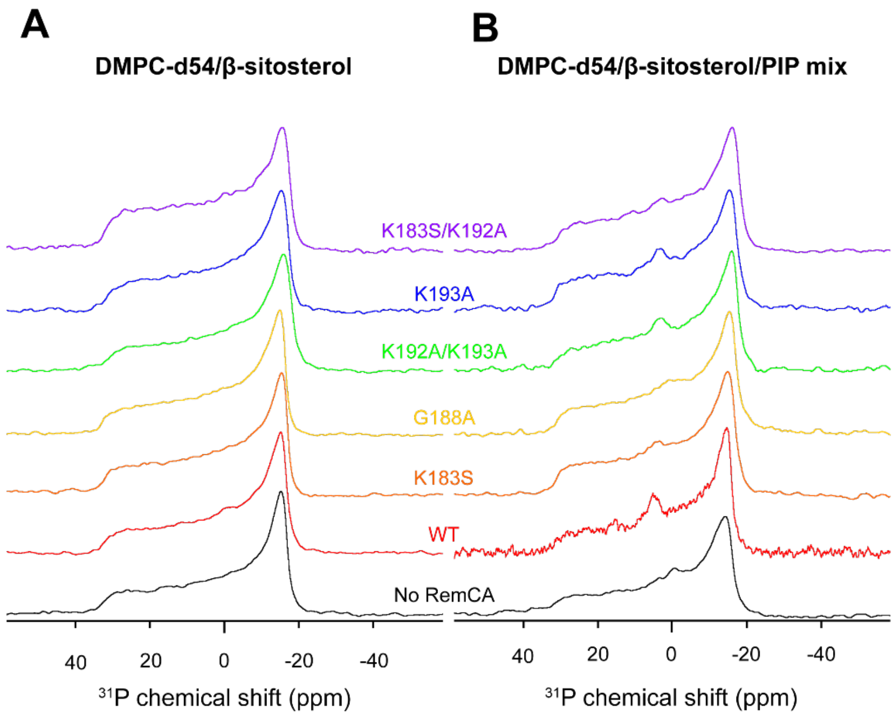

Figure S9. ^{31}P Hahn echo spectra in presence of various RemCA mutants. Liposome compositions are (A) DMPC-d54/β-sitosterol 85/15 and (B) DMPC-d54/β-sitosterol/PIP mix 75/15/10 (molar ratio), at pH=7-8.

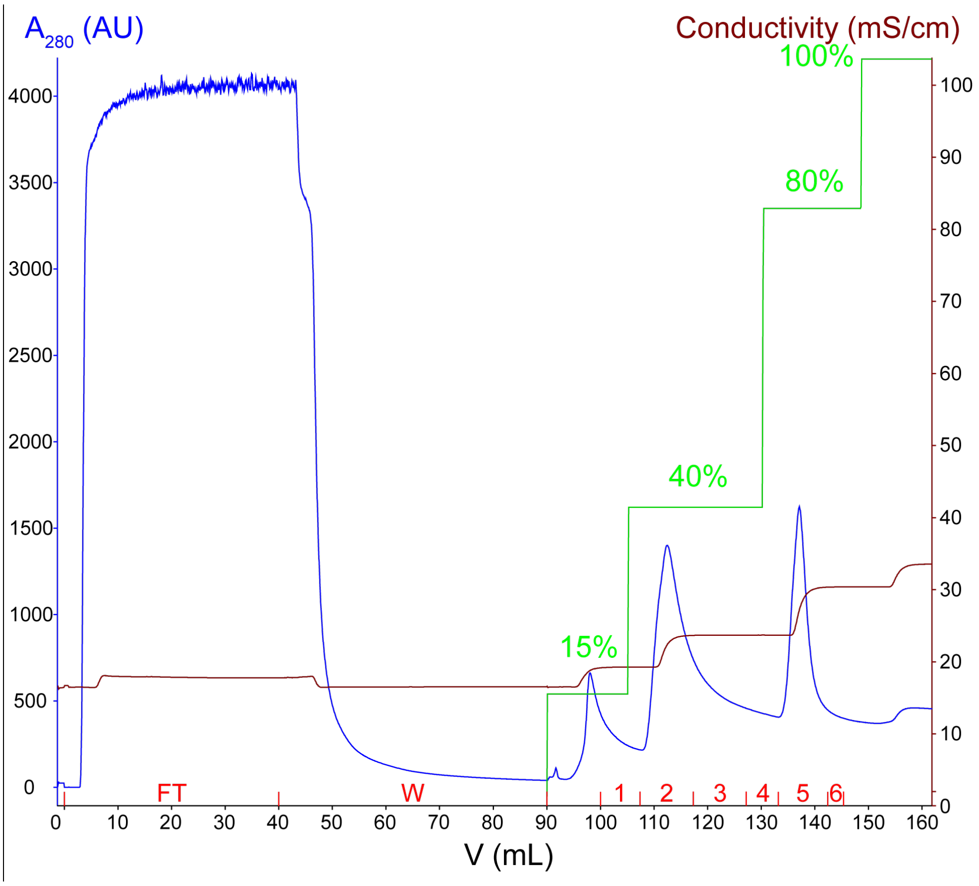

Figure S10. Histrap purification chromatogram of ^{15}N-, ^{13}C-labelled *St*REM1.3. FT: flow-through. W: wash. Blue curve: absorbance at 280 nm in arbitrary units. Brown curve: conductivity (mS/cm). Green curve: elution buffer percentage.

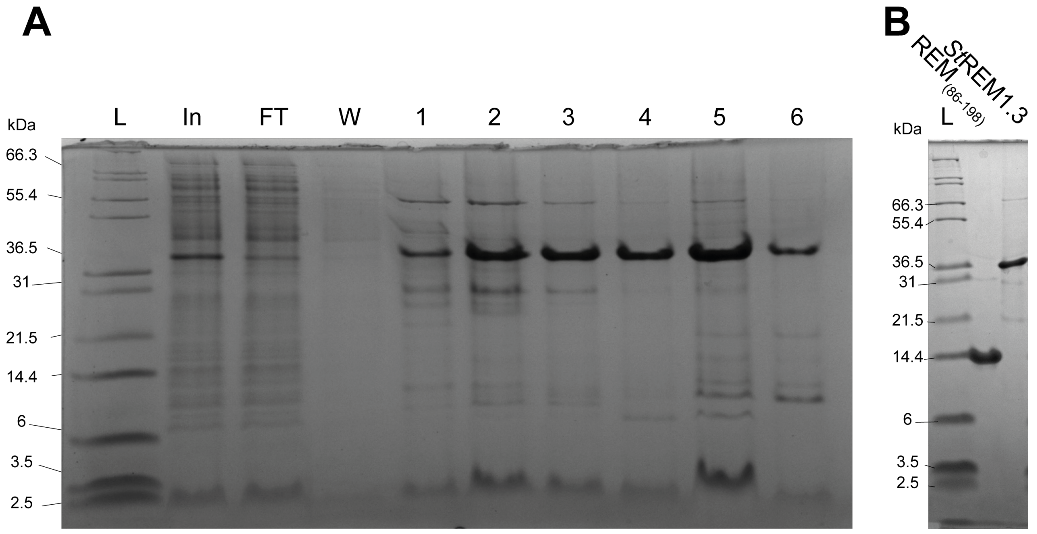

Figure S11. (A) SDS-PAGE of eluted fractions from figure S8. In: sample loaded into the column. 5 and 6 are pooled and purified further. (B) Example of pure protein samples for $REM_{86\text{-}198}$ and *St*REM1.3 analysed by SDS-PAGE. Coomassie-stained gels.

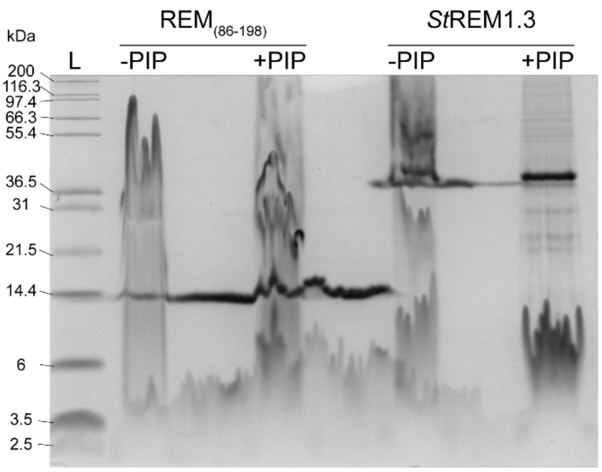

Figure S12. SDS-PAGE of liposomes containing, or not, PIP mix in presence of $REM_{(86\text{-}198)}$ or *St*REM1.3, at pH=7.4. Coomassie-stained gel.

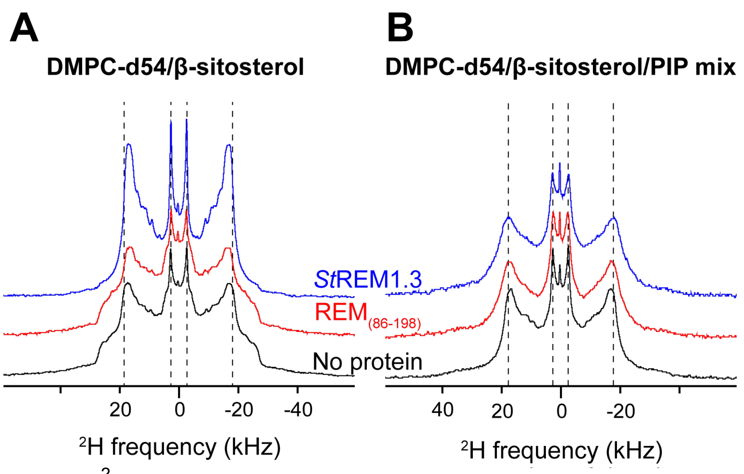

Figure S13. Comparison of ^{2}H quadrupolar spin echo spectra acquired at 298K in absence (black) or presence of $REM_{86\text{-}198}$ (red) or *St*REM1.3 (blue). Inner line pairs indicate the Pake doublet frequency of the terminal CD_3 while outer line pairs indicate the plateau region (usually between positions 2 and 8). Liposome compositions are (A) DMPC-d54/β-sitosterol 85/15 and (B) DMPC-d54/β-sitosterol/PIP mix 75/15/10 (molar ratio), at pH=7.4.

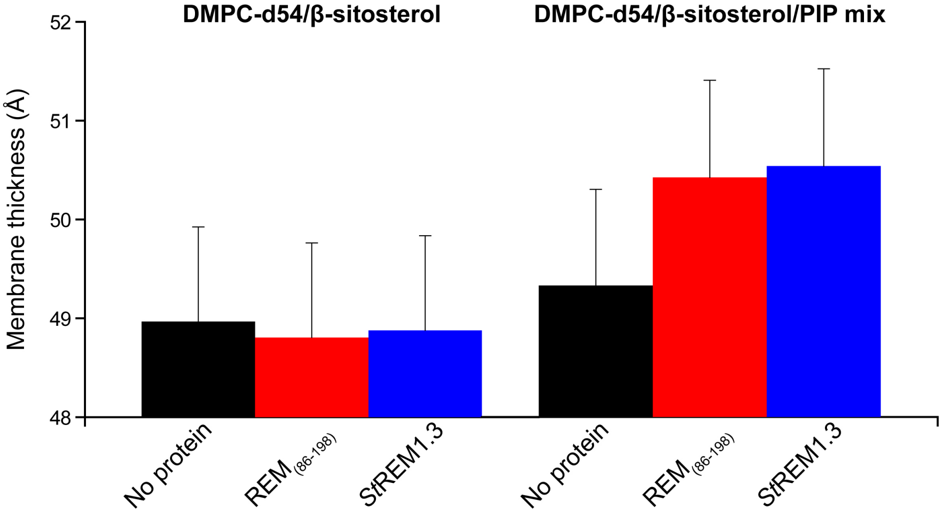

Figure S14. Membrane thickness at 298K calculated from the corresponding $|2{*}S_{CD}|$ (Figure 8). Liposome compositions are DMPC-d54/β-sitosterol 85/15 and DMPC-d54/β-sitosterol/PIP mix 75/15/10 (molar ratio), at pH=7.4. Error bars are assessed as described in Figure S1A, C, E.

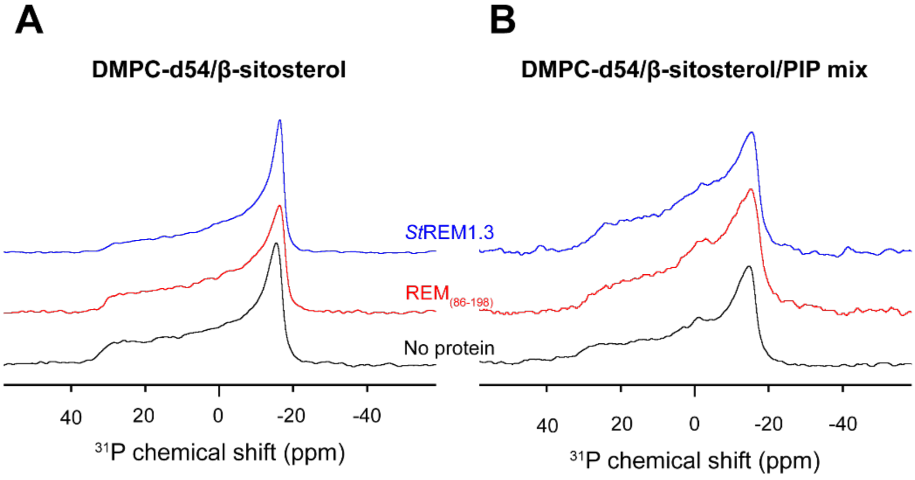

Figure S15. ^{31}P Hahn echo spectra in absence (black) or presence of $REM_{86\text{-}198}$ (red) or *St*REM1.3 (blue). Liposome compositions are (A) DMPC-d54/β-sitosterol 85/15 and (B) DMPC-d54/β-sitosterol/PIP mix 75/15/10 (molar ratio), at pH=7.4.

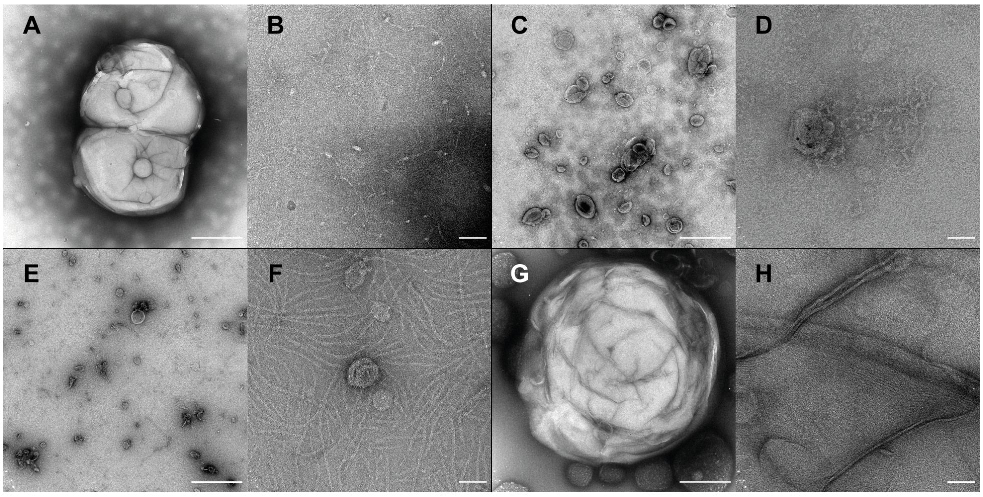

Figure S16. Negative staining electron microscopy of $REM_{86\text{-}198}$ (A-D) and *St*REM1.3 (E-H) in liposomes DMPC-d54/β-sitosterol 85/15 (A, B, E, F) and DMPC-d54/β-sitosterol/PIP mix 75/15/10 (C, D, G, H) (molar ratio), at pH=7.4. Scale bars: (A, C, E, G) 500 nm or (B, D, F, H) 50 nm. Fewer filaments are observed for $REM_{86\text{-}198}$ compared to *St*REM1.3 and the presence of PIP mix seems to reduce their number even more.

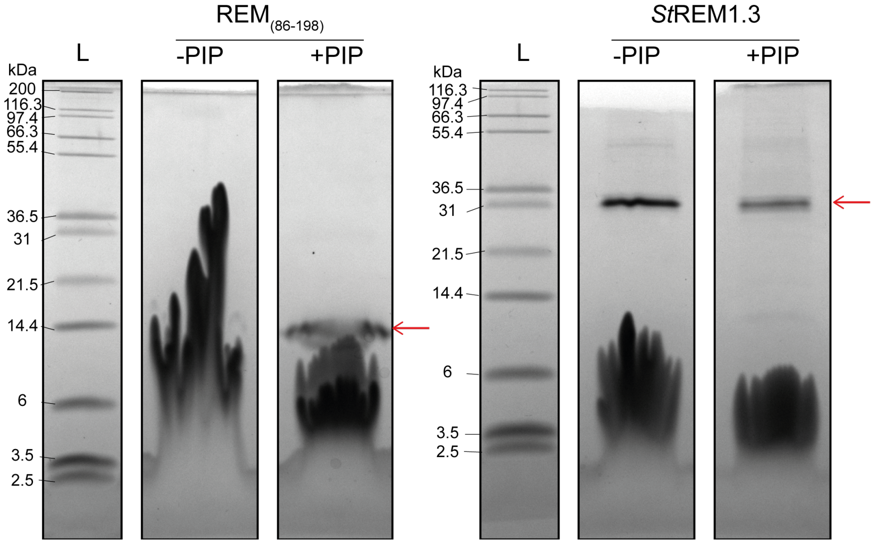

Figure S17. SDS-PAGE of liposomes containing, or not, PIP mix in presence of $REM_{86\text{-}198}$ or *St*REM1.3 when attempting to remove filaments, at pH=7.4. For $REM_{86\text{-}198}$ in liposomes in absence of PIP mix, the protein band might be hidden by the smear but the gel indicates a lack of a band. Red arrows indicate the expected molecular weight of each protein.

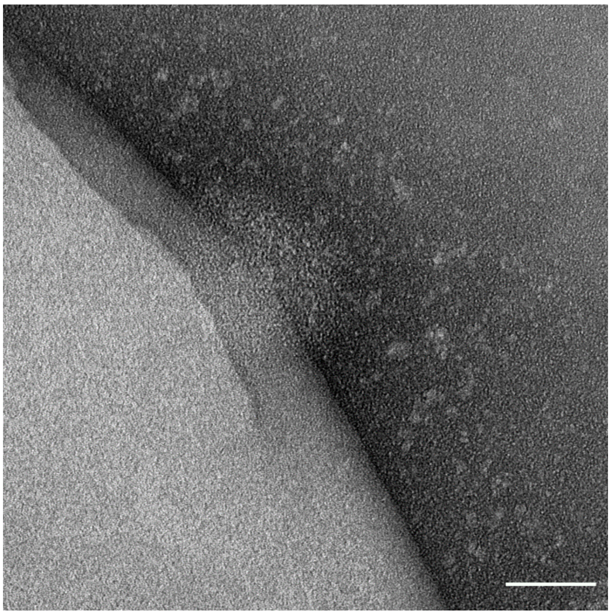

Figure S18. Negative staining electron microscopy of $REM_{86\text{-}198}$ in liposomes without filaments. Liposome composition is DMPC-d54/β-sitosterol/PIP mix 75/15/10 (molar ratio), at pH=7.4.

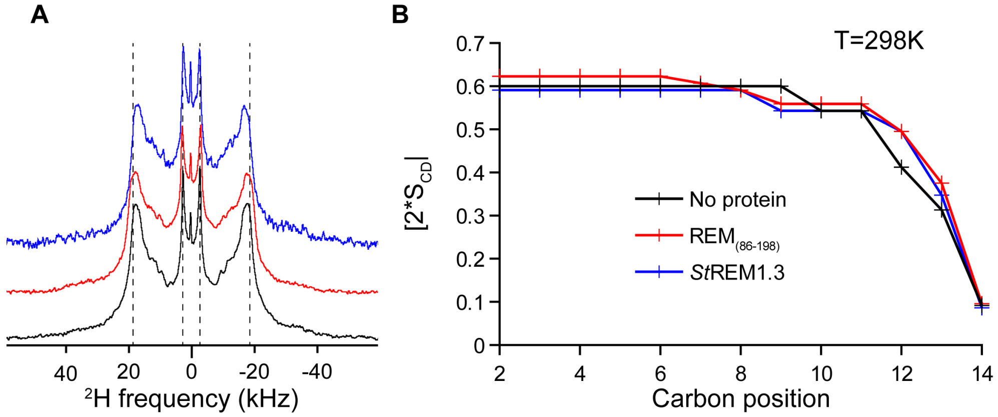

Figure S19. (A) Comparison of ^2H quadrupolar spin echo spectra acquired at 298K in absence (black) or presence of $REM_{86\text{-}198}$ (red) or *St*REM1.3 (blue) without filaments in either case. Inner line pairs indicate the Pake doublet frequency of the terminal CD_3 while outer line pairs indicate the plateau region (usually between positions 2 and 8). (B) Local order parameters $|2{*}S_{CD}|$ as function of carbon position along the acyl chains of DMPC-d54 from spectra in (A). Liposome composition is DMPC-d54/β-sitosterol/PIP mix 75/15/10 (molar ratio), at pH=7.4. Representative error bars for (B) are shown in **Figure S1D.**
